# Efficacy and Safety of Beating Heart Mitral Valve Replacement

**Published:** 2014-04-01

**Authors:** Mohd Lateef Wani, Abdul Gani Ahangar, Shyam Singh, Ifat Irshad, Nayeem ul-Hassan, Shadab Nabi Wani, Farooq Ahmad Ganie, Mohd Akbar Bhat

**Affiliations:** 1 Department of Cardiovascular and Thoracic Surgery, Sher-i-Kashmir Institute of Medical Sciences, Srinagar, Kashmir, India; 2 Lal Ded Hospital, Srinagar, Kashmir, India

**Keywords:** Mitral Valve, Mitral Valve, Heart Arrest

## Abstract

**Background:**

The interest in beating heart surgery is growing since better results can be obtained with this procedure compared to conventional myocardial protection techniques using cardioplegic solutions. This led us to consider mitral valve replacement with beating heart.

**Objectives:**

This study aimed to determine the safety and efficacy of beating heart mitral valve replacement without cross clamp.

**Methods:**

This prospective study was conducted on the patients with isolated mitral valve disease requiring mitral valve replacement according to ACC / AHA guidelines. In this study, 15 patients underwent mitral valve replacement using beating heart technique (Group A) and 15 ones underwent mitral valve replacement using arrested heart technique (Group B). The patients were randomized using block randomization. The data were analyzed using the SPSS statistical software.

**Results:**

Preoperative parameters were comparable in the two groups. Most of the patients in both study groups were in NYHA class III or IV. Postoperatively, however, most of the patients in the two groups were either in NYHA class I or II. No mortality occurred in the beating heart group, while one mortality occurred in the arrested heart group. The results showed a significant difference between the two groups regarding the mean bypass time, mean operating time, mean ICU stay, and mean length of hospital stay.

**Conclusions:**

Beating heart mitral valve replacement is equally safe as the arrested heart technique. Thus, it is recommended as an appropriate alternative to the arrested heart technique for mitral valve replacement.

## 1. Background

The main concern of surgical teams after cardiopulmonary bypass invention has been the deleterious effects of myocardial ischemia. Therefore, ways and means of preventing the harmful effects of myocardial ischemia were taken into account (1). A variety of ways and means was devised, but none was proved to be ideal for achieving optimal results under all clinical circumstances (2).

Interest in beating heart cardiac surgery without aortic cross clamping and, whenever possible, without Cardiopulmonary Bypass (CPB) has increased in the recent years as a means of avoiding the adverse effects of CPB and cross-clamp. Coronary artery bypass surgery has been one of the major beneficiaries of this technique (3-6). Overall, studies have shown better results with beating heart technique than with arrested heart technique. Moreover, duration of ventillatory support, need for transfusion, incidence of postoperative arrhythmias, length of hospital stay, and procedural cost have been positively correlated with the beating heart technique (4-7).

In Kashmir valley, minimally invasive cardiac surgery along with off-pump beating heart CABG, beating heart ASD repair has been performed for over a decade now, resulting in significant reduction in ischemic damage to myocardium and fewer postoperative complications (8). Given the satisfactory results obtained with this approach, the beating heart technique was considered as an option for mitral valve replacement.

## 2. Objectives

We decided to conduct a study to assess the safety and efficacy of beating heart mitral valve surgery compared to arrested heart mitral valve surgery.

## 3. Patients and Methods

The present study was conducted on 30 patients who had isolated mitral valve disease and were subjected to mitral valve replacement. In this study, 15 patients underwent mitral valve replacement using beating heart technique (Group A) and 15 ones underwent mitral valve replacement using arrested heart technique (Group B). The patients were randomized into two groups by computer generated numbers. The same general anesthetic technique with routine arterial and venous monitoring was utilized for both groups. Additionally, mitral valve replacement was performed by the same surgical team. In each patient, the diseased mitral valve was replaced with the mechanical / bioprosthetic valve. The same CPB machine was used in both study groups. Mitral valve was replaced through either sternotomy or right anterolateral thoracotomy.

In Group A, the valve replacement procedure was carried out on beating heart under normothermic conditions. After heparinisation, CPB was established using ascending aorta and bi-caval cannulation. A vent was placed through the right superior pulmonary vein into the left atrium. No vascular cross clamp was placed on the ascending aorta between the arterial perfusion cannula and the cardioplegic cannula. Body temperature was kept at 36°C - 37°C. The maximum flow rate was calculated as per body surface area (2.5 L / Min / m^2^). The mean systemic pressure was maintained above 60 - 80 mmHg. The heart was perfused through the aortic root. No cardioplegia was used. During the procedure, the patients were placed in Trendelenburg position to prevent air embolism. Then, the left atrium was opened and the mitral valve was examined and excised. Chordae sparing technique was used whenever possible. Afterwards, the valve was replaced using prosthesis (Mechanical or Bioprosthetic) through continuous suture technique. After valve replacement, the left atrium was closed in two layers by non-absorbable sutures. We also used aortic and pulmonary vents for removal of air and maintained them until the patients were weaned off bypass. Simultaneously, the lung was briefly inflated to help air removal. Myocardial function was monitored intra-operatively using five-lead electrocardiography (ECG). Arterial blood pressure, central venous pressure, and urine output were also monitored continuously. Arterial blood gas was done every 30 minutes. The patients were weaned off bypass slowly and decannulated as usual after heparin reversal.

In Group B, a vascular cross clamp was placed on the ascending aorta between the arterial perfusion cannula and the cardioplegic cannula. A warm blood cardioplegic solution was administered to stop electrical and mechanical heart activity during diastole. Repeated doses of this solution were administered every 20 - 25 minutes to maintain cardiac arrest. To maintain perfusion under moderate hypothermic conditions, a heat exchanger was used to lower the body temperature to approximately 35°C. Finally, the mitral valve was replaced by the same surgical technique as mentioned above.

The haematocrit level was maintained between 20 - 25% during CPB in both groups. Besides, the pump flow rate was between 2 - 2.5 L / min / m^2^ and the mean arterial pressure was maintained between 60 and 80 mmHg during CPB. The patients were shifted to surgical intensive care unit and were electively ventilated over several hours. They were intensely monitored by intensivists. Oral anticoagulation was started on the 2nd postoperative day with acenocoumarol to maintain an INR of 2 - 3.0. In addition, intravenous antibiotics, a combination of ceftriaxone / sulbactum and amikacin, were administered during the hospital stay.

The follow-up information was collected through telephonic interviews and in the follow-up clinic. The study patients were evaluated regarding the NYHA class and valve-related complications at the time of follow-up. In addition, a complete physical examination, ECG, Chest X-ray, and echocardiography were performed two months after the surgery.

### 3.1. Statistical Analysis

All the data were analyzed using the Statistical Package for Social Sciences (SPSS), version 22. The two groups were compared regarding the NYHA class and its effect on the outcome. Mann-Whitney U test was used to compare the two groups concerning the mean bypass time, mean operation time, mean ICU stay, mean length of hospital stay, and mean postoperative ventilation time.

## 4. Results

Rheumatic heart disease was responsible for 93.3% of cardiac valvular lesion in both study groups. The mean age of the patients in the beating heart group was 42.66 ± 7.1 years, whereas the mean age of those who underwent mitral valve replacement on arrested heart was 43.13 ± 8.5 years (P = 0.723). All the patients were in either NYHA class III or IV preoperatively. However, two months after the operation, a statistically significant improvement was observed in the NYHA class. All the patients were in either class I or II two months after the surgery (Table 1). Most of the patients were operated through right anterolateral thoracotomy and St. Jude mechanical valve was used in most of the patients. The mean bypass time was 108.33 ± 9.9 minutes in the patients who underwent mitral valve replacement on beating heart and 127.93 ± 15.4 minutes in the arrested heart group. The results of Mann-Whitney U test revealed a significant difference between the two groups regarding the mean bypass time (Table 2). The mean aortic cross clamp time was 75.46 ± 10.1 minutes in the patients who underwent mitral valve replacement on arrested heart, whereas cross clamp was not applied in the beating heart group. The absence of cross clamp in the beating heart group eliminated the risk of global myocardial ischemia and its hazards. The mean operating time was 163.20 ± 9.4 and 186.66 ± 19.56 minutes in the beating heart group and arrested heart group, respectively and the difference was statistically significant (Table 2). The patients who underwent mitral valve replacement on beating heart stayed in ICU for a mean duration of 15.66 ± 6.97 hours, while those in the arrested heart group had a mean ICU stay of 22.40 ± 11.12 hours. Furthermore, the patients who underwent mitral valve replacement on beating heart stayed for an average period of 7.06 ± 1.03 days in the hospital post-operatively, whereas the average post-operative hospital stay for the patients who underwent the operation on arrested heart was 8.13 ± 1.99 days. In this study, 1 patient died in the arrested heart group 48 hours after the surgery yielding an immediate mortality of around 7%. However, structural deterioration did not occur in any of our patients. On the other hand, non-structural dysfunction occurred in one of the patients. This patient had mild paravalvular leak at the implant site. Besides, valve thrombosis developed in one patient who had not taken anticoagulants for one week. Thus, she was admitted in the cardiology department and was thrombolized. She behaved well and is now on strict follow-up. In the current study, INR was maintained between 2.5 and 3. None of our patients developed embolism. Bleeding event occurred in one of the patients who developed severe gum bleeding. Also, prosthetic valve endocarditis did not develop in any of our patients and none of them needed reoperation. No valve related mortality was detected in this study. Nonetheless, one of the patients in the arrested heart group died in immediate postoperative period because of low cardiac output syndrome. Additionally, 2 patients had permanent valve related impairment. Also, one of the patients operated on beating heart had paravalvular leak. On the other hand, another patient who was operated on arrested heart had valve thrombosis.

**Table 1.** The Patients' NYHA Class

Preoperative NYHA Class	I	II	III	IV
**Beating heart group**	0	0	10 (66.6%)	5 (33.3%)
**Arrested heart group**	0	0	12 (80%)	3 (20%)
**Post-operative NYHA class**	I	II	III	IV
**Beating heart group**	10 (66.6%)	5 (33.3%)	0	0
**Arrested heart group**	9 (60%)	5 (33.3%)	0	0

**Table 2.** Mean bypass Time (in Minutes) and Mean Operating Time ^a^

Group	No. of Patients	Mean ± SD	Range
**Beating heart group**	15	108.33 ± 9.9	90 - 128
**Arrested heart group**	15	127.93 ± 15.4	95 - 160
**Beating heart group**	15	163.20 ± 9.44	140 - 178
**Arrested heart group**	15	186.66 ± 19.56	140 - 210

^a^ P < 0.001 for both mean bypass time and mean operating time

## 5. Discussion

Chronic rheumatic disease is endemic in the developing world in contrast to the West and remains the most common cause of both mitral stenosis and regurgitation (9). Calcification, particularly at the commisurral edges and occasionally extending posteriorly into the annulus and subvalvular apparatus, is common in later stages of the disease. The mechanism of mitral regurgitation in rheumatic heart disease is type IIIa dysfunction (10-12). Sometimes, anterior leaflet chordal elongation can cause type II dysfunction. Anterior leaflet prolapse and posterior leaflet restriction are also among the most common mechanisms of mitral regurgitation (10). Around one third of the patients with rheumatic heart disease have pure mitral stenosis, while the rest have a combination of mitral stenosis and regurgitation (13).

In order to perform a precise and complete surgical procedure on the heart, it is optimum to have mechanically quiescent heart with bloodless field. These optimal conditions are provided at the cost of global myocardial ischemia (due to cross clamp) and necessitate appropriate myocardial management to limit the damage that would otherwise result from the period of global myocardial ischemia. Damage from a period of ischemia may result in a variable and sometimes prolonged period of both systolic and diastolic dysfunction without muscle necrosis. This condition is now termed as myocardial stunning. The period of ischemia may also result in irreversible damage (myocardial necrosis). Some investigators have obtained information indicating that this can develop after as little as 20 minutes of normothermic ischemia (14, 15). Nayler and Elz stressed the extreme heterogeneity among the cells regarding the progression rate of ischemic damage as well as the rapidity of chain of events of ischemia. They also emphasized the key role of calcium in reperfusion injury (16). In the present study, the mean aortic cross clamp time was 75.46 ± 10.1 minutes in the patients who underwent mitral valve replacement on arrested heart.

All these effects of global myocardial ischemia are absent without a cross clamp. Therefore, operating a patient on beating heart without cross clamp nullifies all the harmful effects of global myocardial ischemia. By nullifying the effects of global myocardial ischemia, these patients have better outcomes compared to those operated on arrested heart with cross clamp. In the present study, 15 patients with mitral valve disease were operated on beating heart without cross clamp. The chief criticism in the literature regarding this approach is the risk of air embolism. According to the literature, the major cerebrovascular events after open heart surgery varied from 1% to 4% (17). In this study, none of our patients had features of air embolism probably because of effective de-airing means used during the procedure. Thompson et al. (18) carried out mitral valve re-operations on 125 patients by utilizing the same approach and noted major cerebrovascular events in two patients (1.6%). Also, no mortality occurred in the beating heart group in this study. However, one of the patients operated on arrested heart died in immediate postoperative period due to low cardiac output. This clearly indicates the advantage of beating heart surgery in mitral valve disease. Although the sample size in our study was too small to draw any conclusion, many researchers have reported a clear mortality advantage on beating heart compared to the arrested heart technique.

In the present study, the mean bypass time was 108.33 ± 9.9 minutes in the patients who underwent mitral valve replacement on beating heart and 127.93 ± 15.4 minutes in those of the arrested heart group and the difference was statistically significant (P = 0.000). Ghosh S et al. (19) reported a mean bypass time of 74.3 minutes in their study. Besides, Morfa GM et al. (20) showed the mean bypass time to be 65 minutes in each group. Gersak B (21) also indicated a statistically significant difference between the two groups regarding the mean bypass time. Similar results were also obtained by Babaroglu S (17). The observed values of total bypass time in our study were well below the highest cutoff value for the total bypass time; i.e., 240 min, which is significantly associated with postoperative morbidity, particularly with postoperative stroke (22).

According to the present study findings, the mean operating time was 163.20 ± 9.4 and 186.66 ± 19.56 minutes in the beating heart group and arrested heart group, respectively and the difference was statistically significant (P = 0.000). Similar results were also obtained by Ghosh S (19), Morfa GM (20), Babaroglu S (17), and Gersak B (21).

In the current study, most of the patients in the beating heart group were extubated within 6 hours of surgery, while it took more than six hours for most of the patients in the arrested heart group to get extubated. However, the difference was not statistically significant (P = 0.244). In the same line, Morfa GM et al. (20) showed no statistically significant difference between the two study groups regarding the ventilation hours. However, Babaroglu S et al. (17) indicated a significant difference between the two groups concerning the post-operative ventilation period. This insignificant difference in our study might be due to the small sample size.

In this study, the patients in both groups were electively ventilated after the surgery and were extubated in the surgical ICU. Afterwards, the patients were shifted to the main cardiac ward. The patients who underwent mitral valve replacement on beating heart stayed in ICU for a mean duration of 15.66 ± 6.97 hours, whereas those in the arrested heart group had a mean ICU stay of 22.40 ± 11.12 hours. The results revealed a statistically significant difference between the two groups concerning the mean ICU stay (P = 0.013). These findings were consistent with those of the studies by Ghosh S (19), Morfa GM (20), Babaroglu S (17), and Gersak B (21). Reduction of ICU stay indirectly reduces the cost of surgery. Hence, this was considered as one of the benefits of beating heart surgery. The time period after the patient was subjected to surgery until discharge from hospital was also considered as one of the variables in this study. The patients who underwent mitral valve replacement on beating heart stayed for an average period of 7.06 ± 1.03 days in the hospital post- operatively, whereas the average post-operative hospital stay for the patients who underwent the operation on arrested heart was 8.13 ± 1.99 days. The study results revealed no significant difference between the two groups in this regard (P = 0.153). Although some researchers have shown a clear difference between the two groups’ means of hospital stay (17), others have revealed no significant differences in this regard (20). In the present study, one patient died in the arrested heart group 48 hours after the surgery, yielding an early mortality of around 7%. The cause of death was low cardiac output. However, none of our patients died in the beating heart group. The study findings indicated a significant difference between the two groups concerning their survival status. Yet, the sample size of the study was too small to draw any conclusions. Similar mortality rates have been noted by other researchers, including Ghosh S (19), Morfa GM (20), and Babaroglu S (17).

Beating heart mitral valve replacement is a safe myocardial protection technique compared to the arrested heart technique. The chief criticism in the literature regarding this approach is the risk of air embolism. However, none of our patients developed the features of air embolism. Another criticism put forward regarding beating-heart mitral valve surgery is the presence of blood at the surgical site which can impair visualization. However, we did not experience problems concerning the visualization of the surgical site because of our venting technique. The advantages of on-pump, beating-heart mitral valve surgery are based on the fact that the heart is operating under a more physiological condition compared to the cardioplegic arrested state, thus eliminating the adverse effects of global myocardial ischemia occurring due to the reperfusion injury. However, the efficacy of this procedure needs to be determined in a larger prospective randomized control trial to compare it with conventional techniques.
